# Modeling and mapping the habitat suitability and the potential distribution of Arboviruses vectors in Morocco

**DOI:** 10.1051/parasite/2021030

**Published:** 2021-04-14

**Authors:** Outammassine Abdelkrim, Boussaa Samia, Zouhair Said, Loqman Souad

**Affiliations:** 1 Laboratory of Microbiology and Virology, Department of Medical Biology, Faculty of Medicine and Pharmacy, Cadi Ayyad University PO Box 7010 40000 Marrakech Morocco; 2 ISPITS-Higher Institute of Nursing and Health Technology 40000 Marrakech Morocco; 3 Ecology and the Environment Laboratory L2E (URAC 32, CNRST ERACNERS 06), Faculty of Sciences Semlalia, Cadi Ayyad University 2390-40080 Marrakech Morocco; 4 Laboratory of Bacteriology–Virology, Avicienne Hospital Military 40000 Marrakech Morocco

**Keywords:** *Aedes* and *Culex*, Arboviruses, Maxent, Morocco, Potential distribution, Habitat suitability

## Abstract

Mosquitoes transmit several agents of diseases and the presence of different species represents a threat to animal and public health. *Aedes* and *Culex* mosquitoes are of particular concern giving their potential vector competence for Arbovirus transmission. In Morocco, the lack of detailed information related to their spatial distribution raises major concerns and hampers effective vector surveillance and control. Using maximum entropy (Maxent) modeling, we generated prediction models for the potential distribution of Arboviruses vectors (*Aedes aegypti, Ae. vexans*, *Ae. caspius, Ae. detritus,* and *Culex pipiens*) in Morocco, under current climatic conditions. Also, we investigated the habitat suitability for the potential occurrence and establishment of *Ae. albopictus* and *Ae. vittatus* recorded only once in the country. Prediction models for these last two species were generated considering occurrence datasets from close countries of the Mediterranean Basin, where *Ae*. *albopictus* is well established, and from a worldwide database for the case of *Ae. vittatus* (model transferability). With the exception of *Ae. vittatus*, the results identify potential habitat suitability in Morocco for all mosquitos considered. Existing areas with maximum risk of establishment and high potential distribution were mainly located in the northwestern and central parts of Morocco. Our results essentially underline the assumption that *Ae. albopictus*, if not quickly controlled, might find suitable habitats and has the potential to become established, especially in the northwest of the country. These findings may help to better understand the potential distribution of each species and enhance surveillance efforts in areas identified as high risk.

## Abstract (fr)

Les moustiques transmettent plusieurs agents de maladies et la présence de différentes espèces représente une menace pour la santé animale et publique. Les moustiques *Aedes* et *Culex* sont particulièrement préoccupants compte tenu de leur compétence de vecteur potentiel pour la transmission des Arbovirus. Au Maroc, le manque d’informations détaillées relatives à leur répartition spatiale soulève des préoccupations majeures et entrave une surveillance et un contrôle efficaces des vecteurs. En utilisant la modélisation de l’entropie maximale (Maxent), nous avons généré des modèles de prédiction pour la distribution potentielle des vecteurs d’Arbovirus (*Aedes aegypti*, *Ae. vexans*, *Ae. caspius*, *Ae. detritus* et *Culex pipiens*) au Maroc, dans les conditions climatiques actuelles. De plus, nous avons étudié l’adéquation de l’habitat pour l’occurrence et l’établissement potentiels d’*Ae. albopictus* et *Ae. vittatus* signalés une seule fois dans le pays. Des modèles de prévision pour ces deux dernières espèces ont été générés en tenant compte des ensembles de données d’occurrence des pays voisins du bassin méditerranéen, où *Ae. albopictus* est bien établi et provient d’une base de données mondiale pour le cas d’*Ae. vittatus* (transférabilité du modèle). À l’exception d’*Ae. vittatus*, les résultats identifient la pertinence potentielle de l’habitat au Maroc pour tous les moustiques considérés. Les zones existantes présentant un risque maximal d’établissement et une distribution à fort potentiel étaient principalement situées dans le nord-ouest et le centre du Maroc. Nos résultats soulignent essentiellement l’hypothèse qu’*Ae. albopictus*, s’il n’est pas rapidement contrôlé, pourrait trouver des habitats convenables et a le potentiel de s’établir, en particulier dans le nord-ouest du pays. Ces résultats peuvent aider à mieux comprendre la répartition potentielle de chaque espèce et à renforcer les efforts de surveillance dans les zones identifiées comme à haut risque.

## Introduction

Over the past few years, arboviruses (arthropod-borne viruses) have (re)emerged at an alarming rate, posing a significant health threat to millions of people worldwide [128]. Dengue virus epidemics (DENV) are responsible for about 50–100 million infections each year [72], Chikungunya virus (CHIKV) is still ongoing periodically since the mid-2000s [123], West Nile virus (WNV) was first introduced to the United States in 1999 and rapidly spread and became endemic throughout North America [131], and most recently Zika virus (ZIKV) quickly spread all over the Western Hemisphere [91] and was declared a Public Health Emergency of International Concern in 2016 [153]. All serve as examples of how explosive and unpredictable arboviral infections outbreaks could be.

Many arboviruses vectored by mosquitoes (Diptera: Culicidae) have expanded their geographic range and managed to achieve greater expansion in areas where they did not exist before. Human movement, global trade, climate change, and availability of susceptible mosquito vectors has increased the introduction of diseases to populations that otherwise would have been safely out of reach [35, 88]. In Morocco, as for the majority of North Africa and Middle East countries, the epidemiological situation of arboviruses remains poorly or even uncharacterized [61]. *Anopheles* species, vectors of malaria, have always been the most studied in Morocco [10, 25, 63, 74–76, 110], while other genera such as *Aedes* and *Culex* remained poorly studied and characterized. Detailed information related to their spatial distribution is scarce, scattered, and rather inaccurate, which hampers effective surveillance and control, especially for the ones representing significant public health threats and listed among *Culicidae* of Morocco or Africa–Mediterranean [141, 142]. This is completely true for the case of *Aedes aegypti* (Linnaeus, 1762)*, Aedes albopictus* (Skuse, 1895)*, Aedes vittatus* (Bigot, 1861)*, Aedes vexans* (Meigen, 1830**)** *, Aedes caspius* (Pallas, 1771)*, Aedes detritus* (Haliday, 1833) and *Culex pipiens* (Linnaeus, 1758), known to be potentially vector competent for Arbovirus transmission (Table 1).

**Table 1:** Overview of the medical importance of certain mosquitos tracked in Morocco.

Species	Period of record in Morocco	Number of times	Reference	Arboviruses transmitted	Reference
*Ae. aegypti*	1916–1997	9	[8, 39, 67, 78, 149]	Zika virus (ZIKV)	[47, 65, 71, 92, 103, 129]
				Chikungunya virus (CHIKV)	[38, 45, 102]
				Dengue virus (DENV)	[9]
				Mayaro virus (MAYV)	[86]
				Uganda S virus (UGSV)	[83]
				Yellow fever virus (YFV)	[95]
*Ae. albopictus*	2016	1	[17]	Zika virus (ZIKV)	[41, 96, 137]
				Chikungunya virus (CHIKV)	[134]
				Dengue virus (DENV)	[32]
				Japanese Encephalitis virus (JEV)	[48]
				Rift Valley fever virus (RVFV)	[33]
				Usutu virus (USUV)	[124]
				West Nile virus (WNV)	[34]
				Yellow fever virus (YFV)	[6, 7]
*Ae. vittatus*	1916	1	[67]	Zika virus (ZIKV)	[50, 51]
				Chikungunya virus (CHIKV)	[49]
				Dengue virus (DENV)	[105]
				Yellow fever virus (YFV)	[70]
*Ae. vexans*	1947–2016	9	[57, 66, 78, 79, 109]	Zika virus (ZIKV)	[58, 69]
				Rift Valley fever virus (RVFV)	[112]
				St. Louis Encephalitis virus (SLEV)	[77]
				Tahyna virus (TAHV)	[107]
				West Nile virus (WNV)	[62]
*Ae. caspius*	1946–2010	59	[57, 150]	Sindbis virus (SINV)	[98]
				Tahyna virus (TAHV)	[122]
				Usutu virus (USUV)	[44]
				Rift Valley fever virus (RVFV)	[139, 145]
				West Nile virus (WNV)	[59, 116]
*Ae. detritus*	1924–2007	53	[39, 81]	Zika virus (ZIKV)	[22]
				Chikungunya virus (CHIKV)	[146]
				Japanese Encephalitis (JEV)	[101]
				Rift Valley fever virus (RVFV)	[97, 139]
				West Nile virus (WNV)	[21]
*Cx. pipiens*	1916–2013	257	[8, 56]	Tahyna virus (TAHV)	[98]
				Japanese Encephalitis virus (JEV)	[127]
				Rift Valley fever virus (RVFV)	[5]
				Sindbis virus (SINV)	[99]
				Usutu virus (USUV)	[36]
				West Nile virus (WNV)	[106]

Recently, ecological niche modeling has been used intensively as the best tool with which to assess, quantify and characterize the risk of mosquitoes’ potential distribution in a defined locality, by relating observed occurrence to environmental data [43, 88]. The approach can provide reliable results even for species with scarce occurrence records [119].

In the context of preventing arbovirus outbreak expansion, knowledge of *Ae. aegypti* and *Ae. albopictus* potential distribution, using ecological niche modeling, has already been shown to help predict the spread of viruses transmitted, such as chikungunya, dengue, and Zika viruses, at both regional and international scales [18, 24, 89]. The work of Kraemer et al. [88] is the best example of the importance of emphasizing the potential threat of vector spread and availability on anticipating arboviruses transmission, especially after the Zika virus emerged in Brazil within a few months of this study’s publication [153].

In the present study, we generated prediction models for the potential distribution of *Ae. aegypti*, *Ae. vexans*, *Ae. caspius*, *Ae. detritus* and *Cx. pipiens* in Morocco. Also, we estimated and evaluated the habitat suitability for the potential occurrence and establishment of *Ae. albopictus* and *Ae. vittatus*, recorded only once in the country. The results produced herein should be considered as a starting point to target and enhance surveillance efforts in areas identified as high risk.

## Materials and methods

### Mosquito records

From the *Culicidae* of Morocco database, tracing back the history of mosquitos in the country from 1916 to 2017, we extracted 9 geo-positioned points for *Ae. aegypti* and *Ae. vexans*, 59 for *Ae. caspius*, 53 for *Ae. detritus,* and 257 for *Cx. pipiens* [140]. Dataset records for *Ae. albopictus* and *Ae. vittatus* were obtained from the Global Biodiversity Information Facility (https://www.gbif.org) and the worldwide database compiled by Kraemer et al. [68, 88, 90]. The downloaded dataset for each species was separately filtered by excluding records with missing latitude or longitude and duplicate records sharing the same coordinates [117]. Over 1550 observed points of *Ae. albopictus* were retained for predictions from close countries of the Mediterranean Basin, where *Ae. albopictus* is well established and was suspected to be the source of the identified population collected in Morocco in 2016 [17]. For *Ae. vittatus*, 429 assembled points were retained and used for predictions at a global scale, given that the species was recorded only once in Morocco without any detailed information regarding its geolocation [141, 142] and also given the scarce occurrence records on the species presence at the regional scale. During model training, the final records were randomly split 20 times into training and testing data in a proportion of 70:30.

### Environmental predictor variables

Any living species can only achieve and maintain its life cycle within a limited range of environmental characteristics. For mosquitos, temperature and precipitation are the most important factors that condition their survival and geographical distribution. Thus, to characterize the current climate conditions, we used data from WorldClim (version 1.4, https://www.worldclim.org). This includes altitude and 19 bioclimatic variables, representing 50 years (1950–2000) of monthly derived temperature and precipitation data, collected from weather stations all over the world at 1 × 1 km (30 arc sec) spatial resolution (Table 2).

**Table 2:** Summary of the environmental variables downloaded.

Environmental variable layers	Signification	Units	Resolution		Reference
			Spatial (km)	Temporal	
Altitude	Elevation above sea level	m	~1 × 1	–	WorldClim^a^
BIO1	Annual mean temperature	°C	~1 × 1	Monthly, 1950–2000	WorldClim
BIO2	Mean diurnal range (mean of monthly (max temp – min temp))	°C	~1 × 1	Monthly, 1950–2000	WorldClim
BIO3	Isothermality (BIO2/BIO7) (× 100)	%	~1 × 1	Monthly, 1950–2000	WorldClim
BIO4	Temperature seasonality (standard deviation × 100)	%	~1 × 1	Monthly, 1950–2000	WorldClim
BIO5	Max temperature of warmest month	°C	~1 × 1	Monthly, 1950–2000	WorldClim
BIO6	Min temperature of coldest month	°C	~1 × 1	Monthly, 1950–2000	WorldClim
BIO7	Temperature annual range (BIO5–BIO6)	°C	~1 × 1	Monthly, 1950–2000	WorldClim
BIO8	Mean temperature of wettest quarter	°C	~1 × 1 km	Monthly, 1950–2000	WorldClim
BIO9	Mean temperature of driest quarter	°C	~1 × 1	Monthly, 1950–2000	WorldClim
BIO10	Mean temperature of warmest quarter	°C	~1 × 1	Monthly, 1950–2000	WorldClim
BIO11	Mean temperature of coldest quarter	°C	~1 × 1	Monthly, 1950–2000	WorldClim
BIO12	Annual precipitation	mm	~1 × 1	Monthly, 1950–2000	WorldClim
BIO13	Precipitation of wettest month	mm	~1 × 1	Monthly, 1950–2000	WorldClim
BIO14	Precipitation of driest month	mm	~1 × 1	Monthly, 1950–2000	WorldClim
BIO15	Precipitation seasonality (coefficient of variation)	%	~1 × 1	Monthly, 1950–2000	WorldClim
BIO16	Precipitation of wettest quarter	mm	~1 × 1	Monthly, 1950–2000	WorldClim
BIO17	Precipitation of driest quarter	mm	~1 × 1	Monthly, 1950–2000	WorldClim
BIO18	Precipitation of warmest quarter	mm	~1 × 1	Monthly, 1950–2000	WorldClim
BIO19	Precipitation of coldest quarter	mm	~1 × 1	Monthly, 1950–2000	WorldClim

a http://www.diva-gis.org/climate.

To select an optimal variable set, a prior modeling test was performed with all of the 20 environmental variables, so as to get a general overview of the variables contributing most to each modeled species. Briefly, the approach consists of running multiple models and each time excluding variables that contribute less or are less informative by comparing model performance with and without the considered variable, which markedly decreases when excluding variables with important contributions and vice versa [84]. To determine the contribution of environmental variables, the Jackknife test option in Maxent was applied [121]. Variables were then submitted to statistical analysis for covariation and collinearity investigation (S1 file and S2 file) using Pearson’s correlation function available in ENMTools package under R system [151, 152]. Variables contributing less with higher correlation (|*r*| < 0.7) to the ones of highest contribution were omitted from the prediction [64]. This process was repeated until left with a set of uncorrelated variables that all had a model contribution [84, 93], which were then used for final predictions (Table 3).

**Table 3:** Correlation matrix of the bioclimatic variables retained for prediction.

	BIO1	BIO10	BIO11	BIO12
BIO1	1	0.225	0.248	0.520
BIO10	0.225	1	0.375	−0.200
BIO11	0.248	0.375	1	0.242
BIO12	0.520	−0.200	0.242	1

### Species distribution modeling

The modeling was carried out using Maxent (Maximum Entropy) software version 3.4.1, which uses an optimization procedure comparing species presence (from occurrence records) with environment characteristics, based on the maximum entropy principle [121]. This machine-learning algorithm, designed to be performed with presence-only record data, has recently gained direct use in various field applications for species distribution modeling, with hundreds of peer-reviewed articles published each year [114]. As the literature recommends, we avoided relying only on the default automatic configuration of Maxent, given increasing debate regarding its use as a black-box, which may not always generate the best results [126, 135]. For each modeled species, we tested a combination of different features (linear, quadratic, product, threshold, and hinge), regularizations multiplier, and used cross-validation to select the optimal settings (S3 file). The Akaike information criterion (AICc) was used to select the optimal combination (the one with minimal AICc value) using NicheA software version 3.0 [93, 125]. Given the lack of occurrence records in some areas, the lack of detailed information on each species distribution range, and the non-availability of absence data, we created a bias file used to fine‐tune background and occurrence point selection in Maxent. For this, we restricted background sampling to a maximum radial distance of no more than 5 km from observation points, using SDMtoolbox [29]. We ran 20 replicates in Maxent for each model and used the mean values to summarize the model predictions results.

### Modeling evaluation

Model performance was evaluated using the partial receiver operating characteristic (pROC) approach, in addition to the area under the curve (AUC). Partial ROC represents a more suitable indicator of statistical significance and allows a better assessment of the niche model predictive ability [120], considering only omission error and proportional area predicted as suitable, and only over a range of omission error deemed acceptable in light of error characteristics of the input data [136]. AUC measures can be misleading and may reflect model accuracy poorly. It weights omission and commission errors equally, does not give information about the spatial distribution of model errors, and summarizes the entire ROC curve, including regions that frequently are not relevant to practical applications [94, 100]. In a partial ROC test, the statistical significance is determined by bootstrap resampling of 50% of testing data, and probabilities are assessed by direct count of the proportion of bootstrap replicates for which the AUC ratio is ≤1.0 [42]. Occurrence datasets and obtained maps were subjected to over 1000 bootstrap iteration analyses, each based on 50% random points resampling, with replacement, and with an omission error threshold of 1% (*p* < 0.01). The pROC statistics test was used using the pROC function available in the NicheToolBox package under R system [118].

## Results

### Modeled habitat suitability

**Figure 1:**
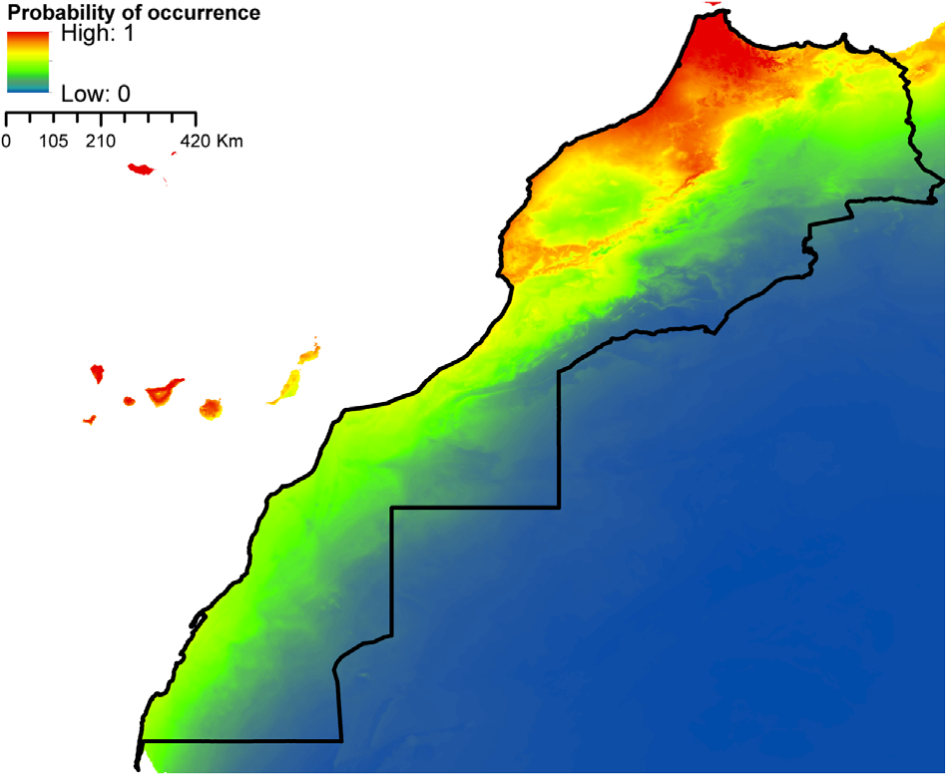
Predicted probability of *Ae. aegypti* occurrence in Morocco.

**Figure 2:**
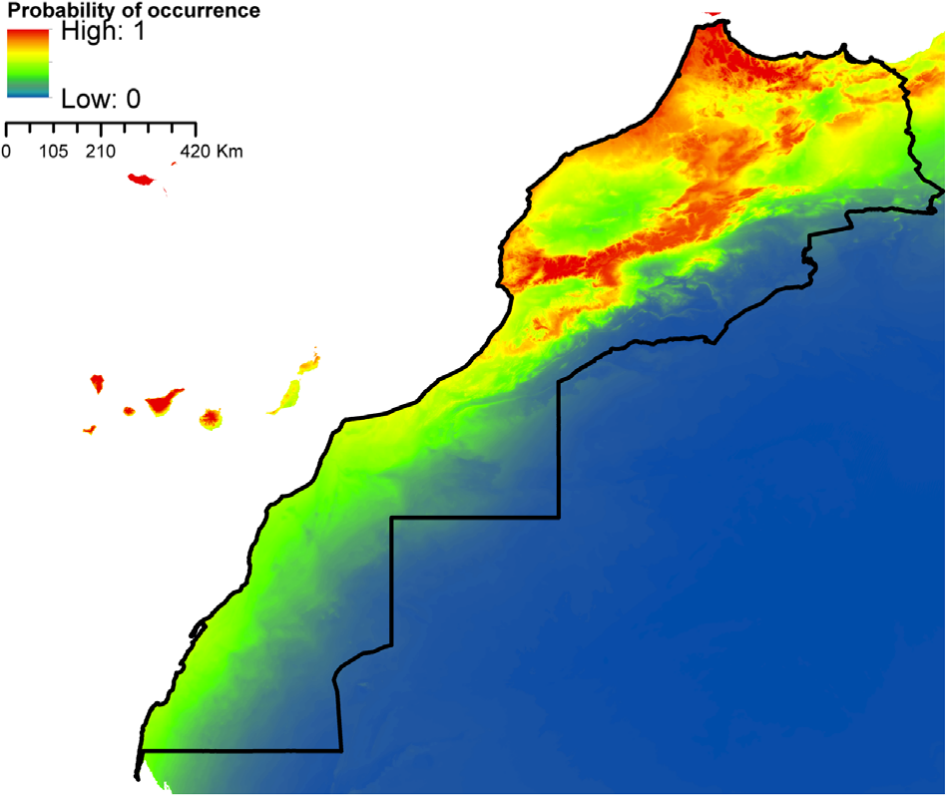
Predicted probability of *Ae. vexans* occurrence in Morocco.

**Figure 3:**
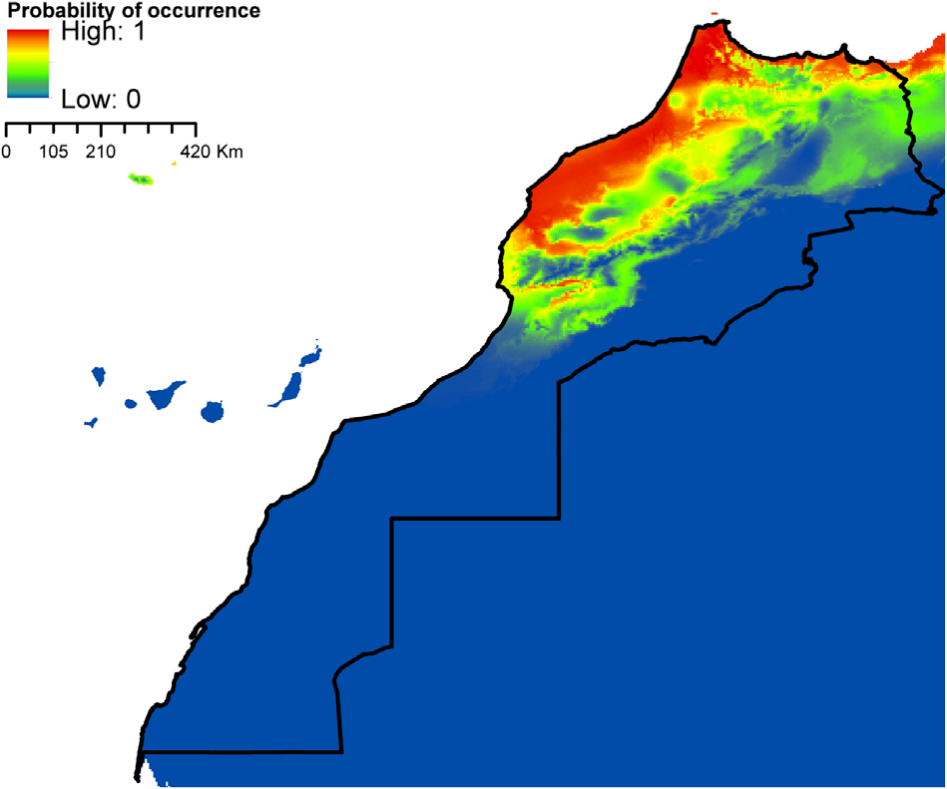
Predicted probability of *Ae. albopictus* occurrence in Morocco.

**Figure 4:**
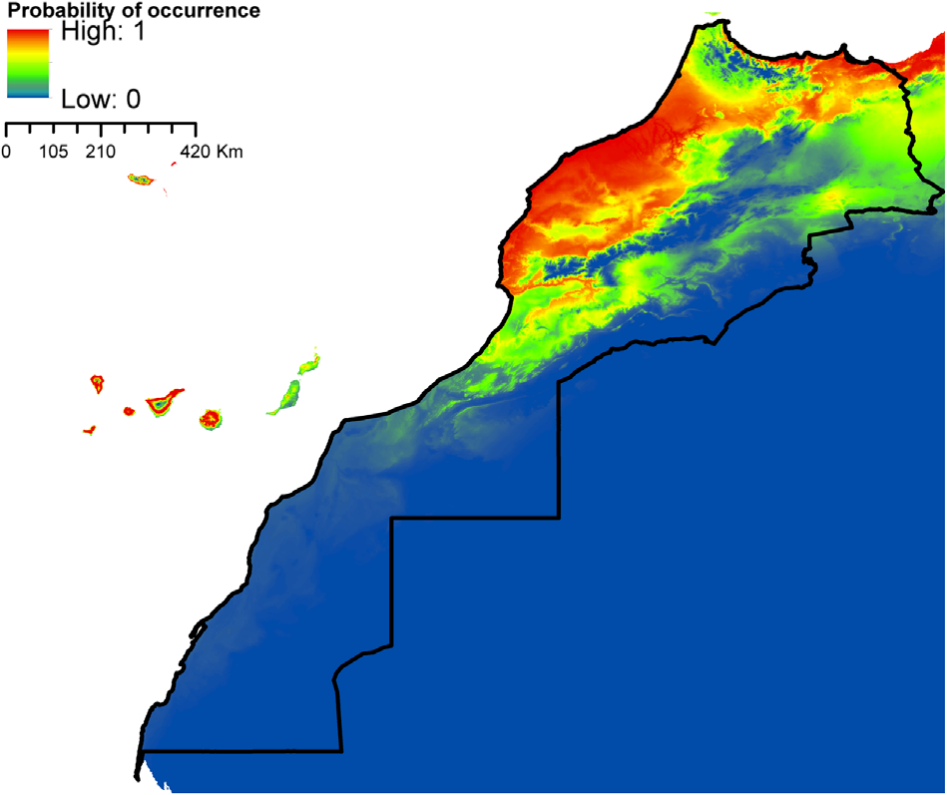
Predicted distribution of *Ae. caspius* in Morocco.

**Figure 5:**
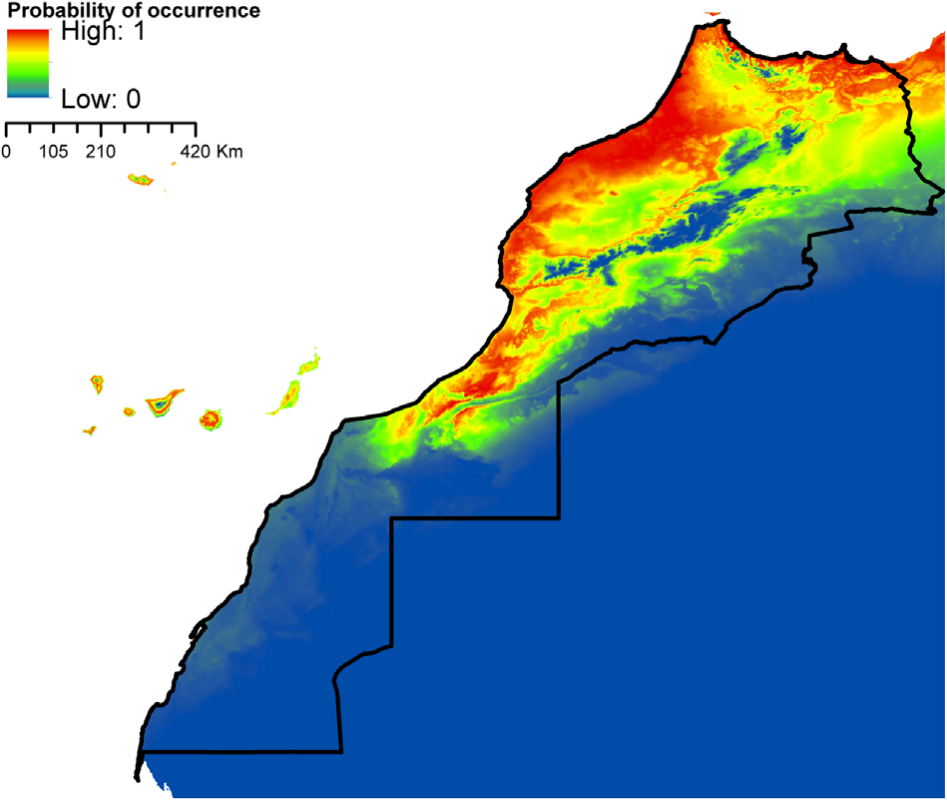
Predicted distribution of *Cx. pipiens* in Morocco.

**Figure 6:**
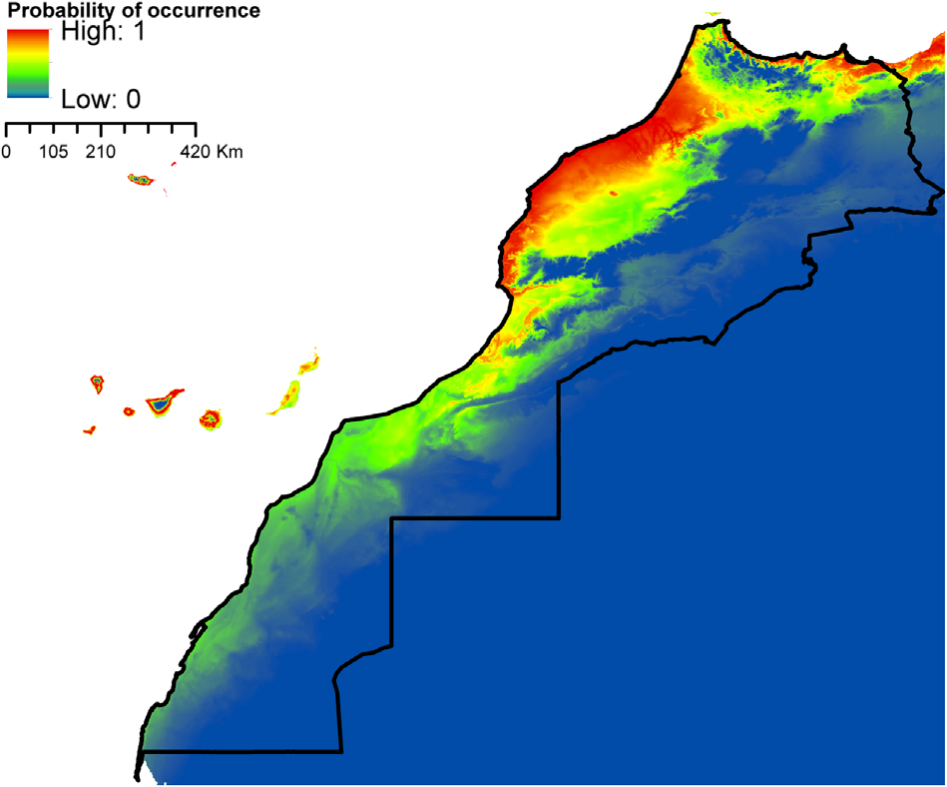
Predicted distribution of *Ae. detritus* in Morocco.

**Figure 7:**
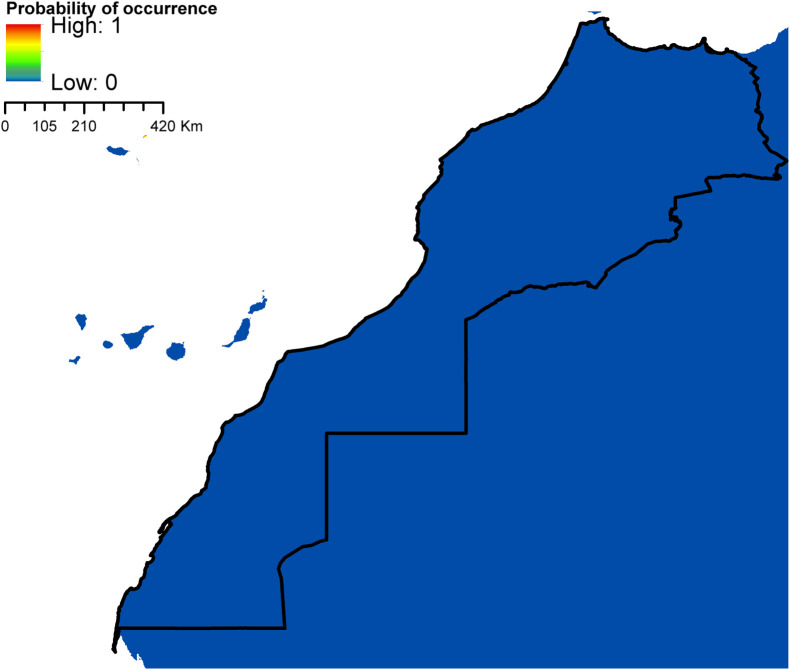
Predicted probability of *Ae. vittatus* occurrence in Morocco.

According to AUC measurement (Table 4), all generated models performed well with AUC values exceeding 0.9 (average over 20 runs) and performed significantly better than random expectations based on the partial ROC test (*p* < 0.01). Maxent predicted widespread environmental suitability for *Ae. aegypti* (Fig. 1 and S4 file) and *Ae. vexans* (Fig. 2, and S5 file) across the country. Areas with the highest risk of potential distribution are essentially located in central parts. *Aedes albopictus* probable risk of occurrence (Fig. 3, S6 file, and S7 file) seems to be highly relevant in areas of the northwest, while the rest of the country was found to be probably unsuitable for establishment. The environmental conditions in Morocco (up to half of the country’s surface area) seem to fit the potential distribution requirement of *Ae. caspius* (Fig. 4 and S8 file) and *Cx. Pipiens* (Fig. 5 and S9 file). Areas classified as highly suitable were primarily located in the center and the northern parts. Moroccan littorals, especially in the north, were modeled at high risk of *Ae. detritus* probable spreading (Fig. 6 and S10 file). For *Ae. vittatus*, the environmental conditions in Morocco seem to be currently unsuitable for potential distribution of this species (Fig. 7, S11 file and S12 file).

**Table 4:** Area under the curve (AUC) values and partial receiver operating characteristic (pROC) ratios summarizing the performance of ecological niche models (average over 20 runs).

Species	Mean AUC*	Bootstrap iterations	pROC ratio				
			Minimum	Maximum	Mean	Median	*p* < 0.01
*Ae. aegypti*	0.924 ± 0.035	1000	1.77	2.00	1.88	1.87	0***
*Ae. albopictus*	0.961 ± 0.002	1000	1.89	1.94	1.92	1.92	0***
*Ae. vexans*	0.945 ± 0.023	1000	1.81	2.00	1.90	1.90	0***
*Ae. vittatus*	0.951 ± 0.013	1000	1.74	1.75	1.74	1.74	0***
*Ae. caspius*	0.988 ± 0.006	1000	1.64	1.88	1.68	1.65	0***
*Ae. detritus*	0.993 ± 0.003	1000	1.75	1.96	1.80	1.76	0***
*Cx. pipiens*	0.984 ± 0.001	1000	1.41	1.70	1.60	1.62	0***

* 0.5 (random) < AUC < 1 (perfect).

*** Highly significant.

### Variable importance

By investigating the relative contribution of the bioclimatic variables used, we were able to identify which of the variables most influences the predictions (Table 5). The mean temperature of the coldest quarter (BIO11) was the most informed variable for *Ae. detritus*, *Ae. caspius* and *Cx. pipiens* potential distribution, followed by the annual mean temperature (BIO1) as the second most contributing. In the case of *Ae. vittatus* and *Ae. aegypti*, BIO1 appears to provide the most useful information contributing with 43.9% and 47.4%, respectively. Concerning *Ae. albopictus* and *Ae. vexans* potential prediction, the mean temperature of the warmest quarter (BIO10) was yielded as the most dependent variable.

**Table 5:** Main contribution of the environmental variables used for modeling.

Environmental variable layers	Permutation importance (%)						
	*Ae. aegypti*	*Ae. albopictus*	*Ae. vexans*	*Ae. vittatus*	*Ae. detritus*	*Ae. caspius*	*Cx. pipiens*
Annual mean temperature (BIO1)	43.9	25	4.2	47.4	20.6	29.2	35
Mean temperature of warmest quarter (BIO10)	17.9	31.2	76.6	9.3	9.3	7.3	15.6
Mean temperature of coldest quarter (BIO11)	1.8	23.3	3.5	35.1	67.5	49.8	47.6
Annual precipitation (BIO12)	36.3	20.6	15.6	8.2	2.5	13.7	1.8

### The one-variable response curves

**Figure 8:**
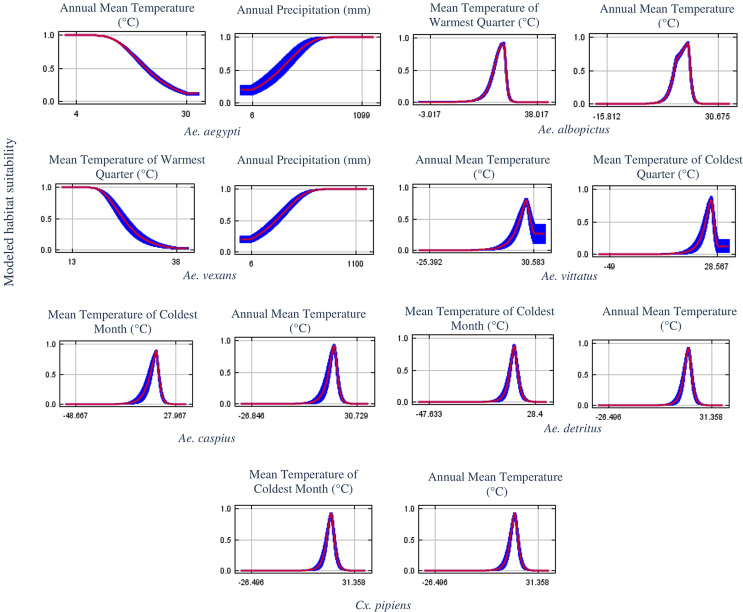
Response curves (for most contributing variables) for the one-variable-models indicating the environmental limits for each vector.

The one-variable response curve generated by Maxent is a powerful tool that helps define the modeled habitat suitability requirement for the species considered, depending on only one variable each time (Fig. 8). In terms of successful establishment thresholds, *Ae. aegypti* is modeled to find suitable conditions in areas where the annual mean temperature is below 25 °C (optimum from 5 °C to 10 °C) with annual precipitation of at least 200 mm (optimum < 600 mm). A similar amount of precipitation with a mean temperature of the warmest quarter of no more than 25 °C (optimum from 10 °C to 17 °C) seems necessary for successful establishment of *Ae. vexans* in the country. In the case of *Ae. albopictus*, areas with mean temperature of the warmest quarter ranging from 17 °C to 27 °C and annual mean temperature of 11–20 °C were modeled as suitable. *Aedes vittatus* is modeled to be potentially present in regions with an annual mean temperature and a mean temperature of the coldest quarter ranging from 20 °C to 30 °C. For *Ae. detritus*, *Ae. caspius* and *Cx. Pipiens* predicted distribution, it seems to highly depend on mean temperature of the coldest quarter and annual mean temperature ranging from 10 °C to 20 °C.

## Discussion

In the present study, we generated prediction models for the potential distribution of the well-known disease vectors *Ae. aegypti*, *Ae. vexans*, *Ae. caspius*, *Ae. detritus,* and *Cx. pipiens* in Morocco. Also, we estimated and evaluated the habitat suitability for the potential occurrence and establishment of *Ae. albopictus* and *Ae. vittatus*, recorded only once in the country.

Knowledge of the current distribution of these mosquito species can be of great value in identifying the areas at risk of probable associated arboviruses expansion. Specifically, the habitat suitability map generated herein can help predict where these species could become established. Also, to focus attention on areas where surveillance could be prioritized, especially where entomological reporting remains poor or where the vector is currently present but has yet to fulfill its potential fundamental niche.

With the exception of *Ae. vittatus*, a species in which the current environmental conditions in the country were modeled unsuitable, the north and central parts of Morocco appeared to be the areas at high risk. Importantly, they were modeled suitable for six species out of seven (*Ae. aegypti*, *Ae. vexans*, *Ae. caspius*, *Cx. pipiens*, *Ae. detritus,* and *Ae. albopictus*). For the southern parts, it seems that conditions might be currently suitable for potential distribution of only *Ae. aegypti* and *Ae. vexans*.

Overall, the risk of establishment of mosquitoes in the country can be classified into two main categories: species with high (*Ae. aegypti*, *Ae. vexans, Cx. pipiens, Ae. caspius, Ae. detritus,* and *Ae. albopictus*) and low (*Ae. vittatus*) probability of establishment. This difference in their potential distribution may reflect unique environmental requirements for each species such as larval habitat.

The minimum temperature or the mean temperature of the coldest quarter were identified as critical factors determining the presence of *Ae. aegypti* in multiple studies [52, 87]. Temperatures ranging from 4 °C to 10 °C were estimated to be the minimum temperature threshold for *Ae. aegypti* [143]. This is consistent with our findings as Maxent predicted suitable conditions in areas where the annual mean temperature is below 25 °C. It is worth mentioning here that the model developed for *Ae. aegypti* (*Ae. vexans* as well) using small sample sizes should be interpreted with caution as the prediction only identifies regions that have similar environmental conditions to the points used, and do not represent actual limits to the range of the species [119]. For example, *Ae. aegypti* was modeled to find suitable conditions in areas where the annual mean temperature is below 25 °C (optimum from 5 °C to 10 °C), according to the one-variable response curve generated by Maxent, an optimum which seems very low for a largely non-temperate species. *Aedes aegypti* is usually known to be tolerant to high temperatures and can be viable following exposure to temperatures up to 40 °C [27], but cannot resist low temperatures. Larval survival requires a temperature higher than 10 °C [144] and prolonged exposure of eggs to temperatures below 10 °C has been fatal [40].

According to our prediction results, *Ae. albopictus* was modeled to find suitable conditions in areas with a mean temperature of the warmest quarter ranging from 17 °C to 27 °C and annual mean temperature of 11–20 °C. This is completely in accordance with the commonly known environmental limits announced by the European Centre for Disease Prevention and Control (ECDC), regarding the successful establishment thresholds of this species in Europe. According to their findings, *Ae. albopictus* could occur in locations where the mean annual temperature exceeds 11 °C (required condition for mosquito activity and survival), a summer temperature of 25–30 °C, mean temperature of the coldest quarter >0 °C, and annual rainfall of at least 500 mm (pre-requisite for aquatic habitats availability and maintenance). However, reports also indicated that this species can successfully establish under lower mean temperatures (5–28.5 °C) and annual precipitation not exceeding 290 mm [54, 108].

Kraemer et al. [88] and Kamal et al. [85] were the first to predict the global potential distribution of *Ae. aegypti* and *Ae. albopictus* using ecological niche modeling. Accordingly, the northwest Atlantic coast of the country was predicted at-risk of *Ae. aegypti* and *Ae. albopictus* potential distribution, which is consistent with our results. It is worth mentioning here that in both studies no points were included from Morocco or North Africa. In addition, our study placed other areas at risk of *Ae. aegypti* distribution including southwestern Morocco where no previous observed occurrence records were available. Either the species exists here but is not yet documented or it is currently absent but the environmental conditions are suitable for possible introduction in the future. Either way, these areas are worth being intensively monitored as soon as possible, especially after the species has recently emerged in North Mauritania and Egypt, after years of presumed absence [3, 113]. Of note, the areas where the species was notified, in North Mauritania and Egypt, share similar environmental conditions with southern Morocco, according to the Köppen–Geiger Climate Classification [14].

*Aedes caspius*, *Cx. pipiens* and *Ae. detritus* predicted distribution in Morocco seems to highly depend on the mean temperature of the coldest quarter and annual mean temperature ranging from 10 to 20 °C. In Roiz et al. [132], a study evaluating climatic effects on mosquito abundance in Mediterranean wetlands using long-term series of mosquito abundance data (2003–2012), the mean temperature was positively related to *Cx. pipiens* and *Ochlerotatus caspius* (*Ae. caspius*) abundances. Also, Ewing et al. [60] demonstrated that increases in mean annual temperature and amplitude of seasonal temperature fluctuations will increase the abundance of temperate mosquitoes (*Cx. pipiens*) in the United Kingdom in the coming years.

*Aedes caspius* potential distribution showed high suitability across the northwestern and northeastern sides of the country, essentially at low altitudes. *Aedes caspius* is a species with a very wide Palearctic distribution; it stretches from Europe to central Asia, and from Egypt to Morocco [55, 130]. It is a very well-represented species in the Mediterranean Basin, mainly along the coast; it has been reported in Italy [147], Belgium [26], France [13, 37], and Spain [73]. In Morocco, the species was mainly collected in coastal and relatively more humid regions. Suitable habitats for *Ae. detritus* are currently limited to the northwestern part of the country, especially along the coastline. Indeed, the species showed a similar distribution pattern in Europe as it is found all over the European coastlines, e.g. in the United Kingdom [23], Italy [104], Belgium [26], France [28], and Spain [133]. It is a common Palearctic species that is more abundant in southern and dry regions [15]. In North Africa, the species has been detected in Egypt [1], Tunisia [16], Algeria [111], etc. In Morocco *Ae. detritus* is very well represented on the littoral zones, where it is found on a fairly regular strip from Tangier to Tantan (the Atlantic coast), and on a less regular strip from Tangier to Saïdia (the Mediterranean coast) [140]. According to our model predictions, up to half of the country’s surface area seems to be suitable for *Cx. pipiens* potential distribution. Areas classified as highly suitable were primarily located in the central and the northern parts. *Culex pipiens* is a very common and ubiquitous species in Morocco, [2, 20]. In temperate regions, particularly in the Mediterranean basin, *Cx. pipiens* is recognized as one of the most widespread cosmopolitan species [5, 30]. The species also dwells in the temperate regions of Africa, Asia, Australia, Europe, North and South America [80].

Versteirt et al. [148] previously identified the current geographic distribution of *Ae. caspius*, *Ae. detritus* and *Cx. pipiens* in Europe and countries surrounding the Mediterranean Basin, including Morocco. According to their findings, *Ae. caspius* and *Ae. detritus* are predicted with high probability on the Atlantic coast of Morocco. By contrast, *Cx. pipiens* was predicted to be highly distributed in areas with more temperate climatic conditions such as the Mediterranean Sea coastline north of Morocco. Our models yielded similar results of habitat suitability for *Ae. caspius* and *Ae. detritus* on the Atlantic coast and *Cx. pipiens* on the Mediterranean Sea coastline but recognized different distributional patterns across the rest of the country. Reasons for such disagreement may be essentially the non-appropriate choice of explanatory variables used for prediction in their study: temperature and vegetation, annual amplitude of night time temperature, variance in night time temperature, variance in the enhanced vegetation index, phase of the annual night time temperature cycle, and maximum of the enhanced vegetation index, which resulted in substantial underestimation of habitat suitability. It is known that temperature and precipitation are the most important factors that condition mosquito survival and distribution, and predictions built with these variables usually produce more realistic results [31, 46, 82, 85, 88].

*Aedes vexans* is considered a nuisance species in central Europe and the Mediterranean region [19]. The species is also indigenous to North America as it is found throughout the United States and southern Canada [115]. In North Africa, the species has been detected in Mauritania, Algeria, Tunisia, and Morocco [140, 141].

Versteirt et al. [148] also identified the current geographic distribution of *Ae. vexans* in Europe and the Mediterranean Basin. Accordingly, *Ae. vexans* is predicted with a very low probability in Morocco. Our model prediction yielded different results of habitat suitability. Reasons for such disagreement may be the non-appropriate choice of explanatory variables used for prediction in their study (as previously discussed), and also the absence of points used from Morocco or other north African countries.

The current distribution of *Ae. vittatus* includes tropical and subtropical areas in Asia and Africa. In Europe, the species is restricted to the occidental Mediterranean region comprising Italy, France, Spain, and Portugal [53, 138].

Giving the potential suitability of the country for the occurrence and distribution of Arboviruses vectors, there is an urgent need to undertake and enhance periodic surveillance campaigns in areas currently considered at high risk. This is particularly important as it was demonstrated recently that *Ae. albopictus* identified in Rabat in 2016 [17] is competent for not only Zika virus transmission but also for many Arboviruses: Dengue, Chikungunya, and Yellow fever viruses [4]. Moreover, multiple cases of some imported arboviruses have been described recently in Morocco, especially Dengue [11] and Chikungunya [12]. With a suitable environment, viremic travelers caring viruses, and the potential wild distribution of known competent vectors, all key elements for potential outbreaks are present.

However, we cannot deny that there are some limits surrounding our study, as the case with every study forecasting habitat suitability or the potential distribution of any living species (Ogden 2017). Specifically, the models emphasize climate (e.g. macroclimate) as the key driver of mosquito distributions. The suitable habitats are modeled based on the assumption that there will not be any dispersed limitation encountered by the species. The impact of biological interactions, such as the presence of potential competitors or predators in the new predicted habitats, is also neglected in our models. Therefore, our prediction is an ideal state and should be considered as a starting point to target and enhance surveillance efforts in areas identified as high risk.

## Supplementary materials

Supplementary material is available at https://www.parasite-journal.org/10.1051/parasite/2021030/olm *S1 File*. Correlation matrix results of the environmental variables collinearity test. *S2 File*. Correlation matrix Heatmap. *S3 File*. Evaluation metrics of the Maxent model runs using different parameters and combinations. *S4 File*. *Ae. aegypti* prediction model with occurrence points. *S5 File*. *Ae. vexans* prediction model with occurrence points. *S6 File*. Modeling results for *Ae. albopictus* in Mediterranean Basin countries. *S7 File*. *Ae. albopictus* prediction model with occurrence points. *S8 File*. *Ae. caspius* prediction model with occurrence points. *S9 File*. *Cx. pipiens* prediction model with occurrence points. *S10 File*. *Ae. detritus* prediction model with occurrence points. *S11 File*. Global potential distribution of *Ae. vittatus*. *S12 File*. *Ae. vittatus* prediction model with occurrence points.
