# Supplementary figures and images for: Modeling and mapping the habitat suitability and the potential distribution of Arboviruses vectors in Morocco
Source: Parasite. 2021 Apr 14;28:37. doi: 10.1051/parasite/2021030 (PMC8051322; doi:10.1051/parasite/2021030)

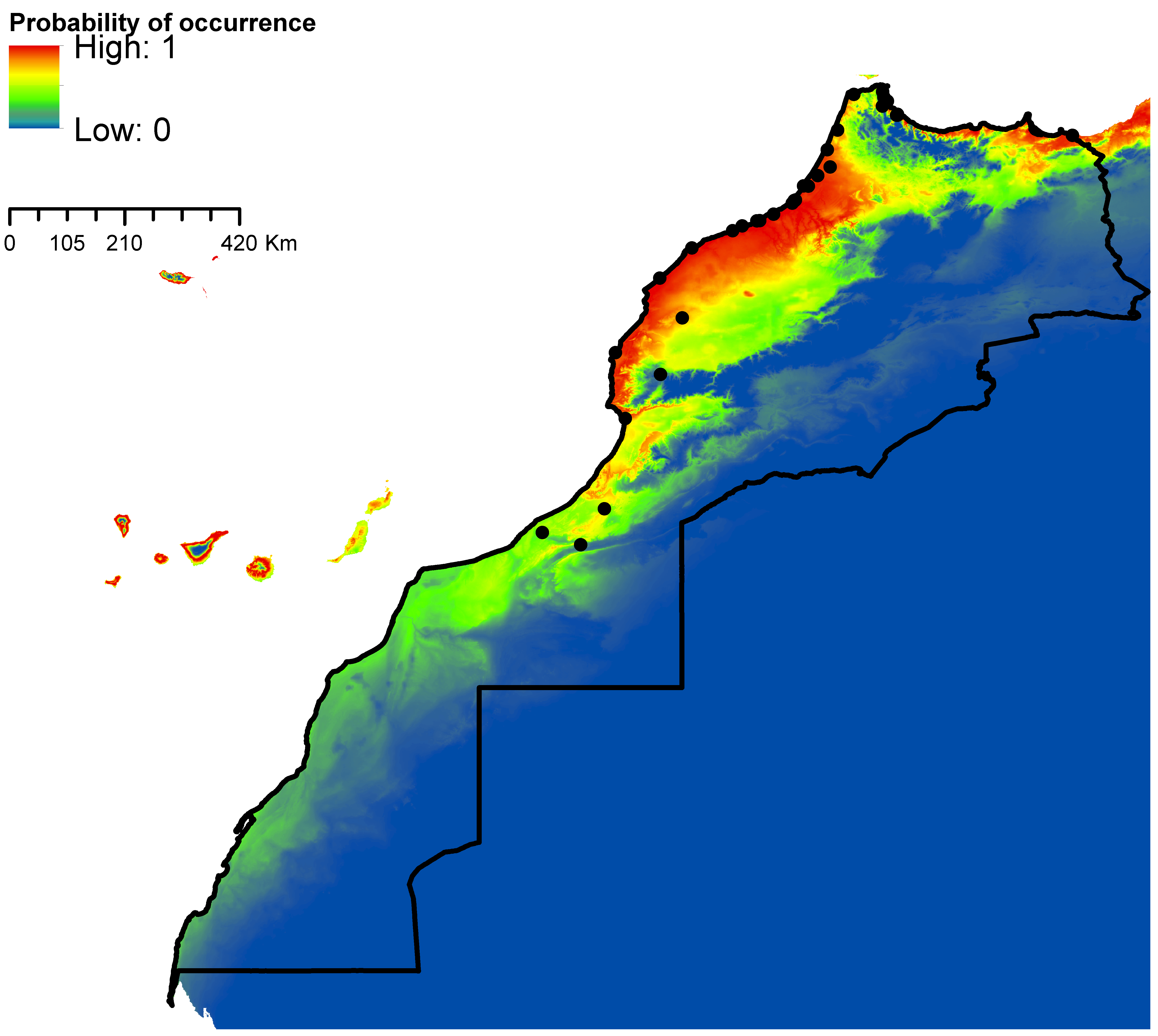

Supplement: Supplementary file 1 — S1 File. Correlation matrix results of the environmental variables collinearity test. S2 File. Correlation matrix Heatmap. S3 File. Evaluation metrics of the Maxent model runs using different parameters and combinations. S4 File. Ae. aegypti prediction model with occurrence points. S5 File. Ae. vexans prediction model with occurrence points. S6 File. Modeling results for Ae. albopictus in Mediterranean Basin countries. S7 File. Ae. albopictus prediction model with occurrence points. S8 File. Ae. caspius prediction model with occurrence points. S9 File. Cx. pipiens prediction model with occurrence points. S10 File. Ae. detritus prediction model with occurrence points. S11 File. Global potential distribution of Ae. vittatus. S12 File. Ae. vittatus prediction model with occurrence points. [file parasite-28-37-olm.zip › parasite200169-olm/S10 File.tif]

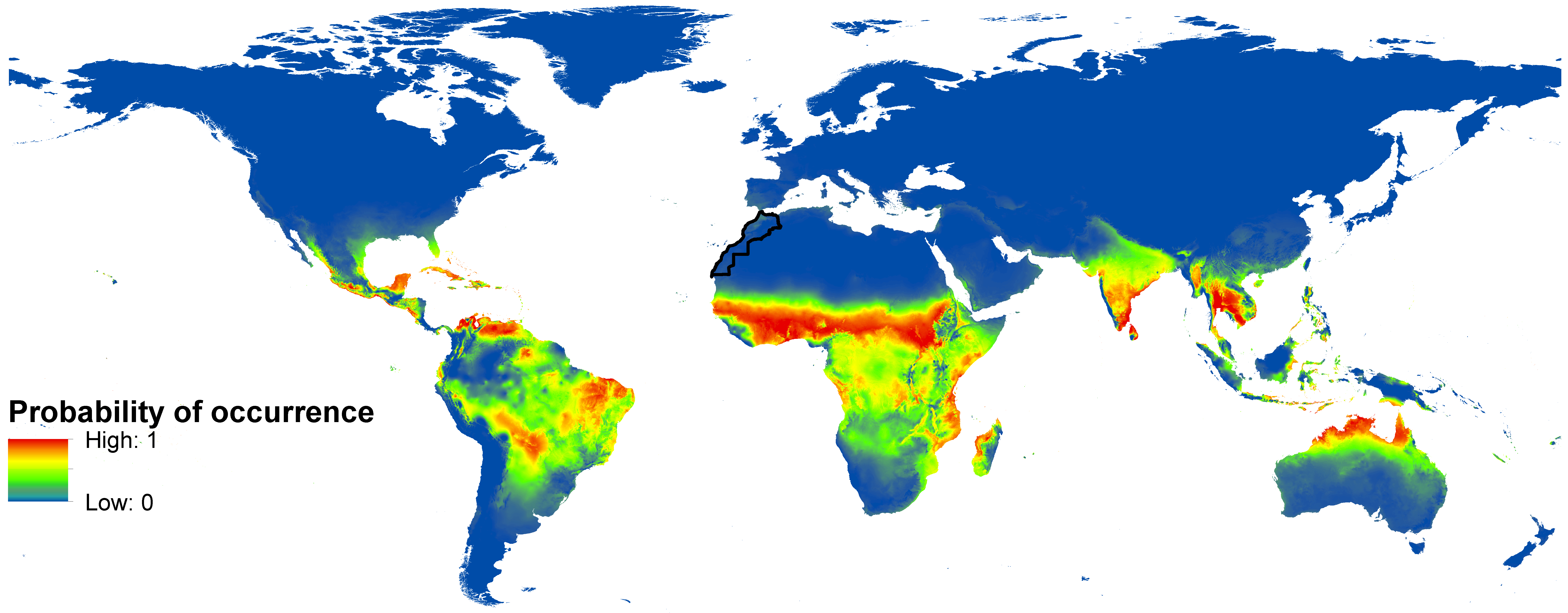

Supplement: Supplementary file 1 — S1 File. Correlation matrix results of the environmental variables collinearity test. S2 File. Correlation matrix Heatmap. S3 File. Evaluation metrics of the Maxent model runs using different parameters and combinations. S4 File. Ae. aegypti prediction model with occurrence points. S5 File. Ae. vexans prediction model with occurrence points. S6 File. Modeling results for Ae. albopictus in Mediterranean Basin countries. S7 File. Ae. albopictus prediction model with occurrence points. S8 File. Ae. caspius prediction model with occurrence points. S9 File. Cx. pipiens prediction model with occurrence points. S10 File. Ae. detritus prediction model with occurrence points. S11 File. Global potential distribution of Ae. vittatus. S12 File. Ae. vittatus prediction model with occurrence points. [file parasite-28-37-olm.zip › parasite200169-olm/S11 File.tif]

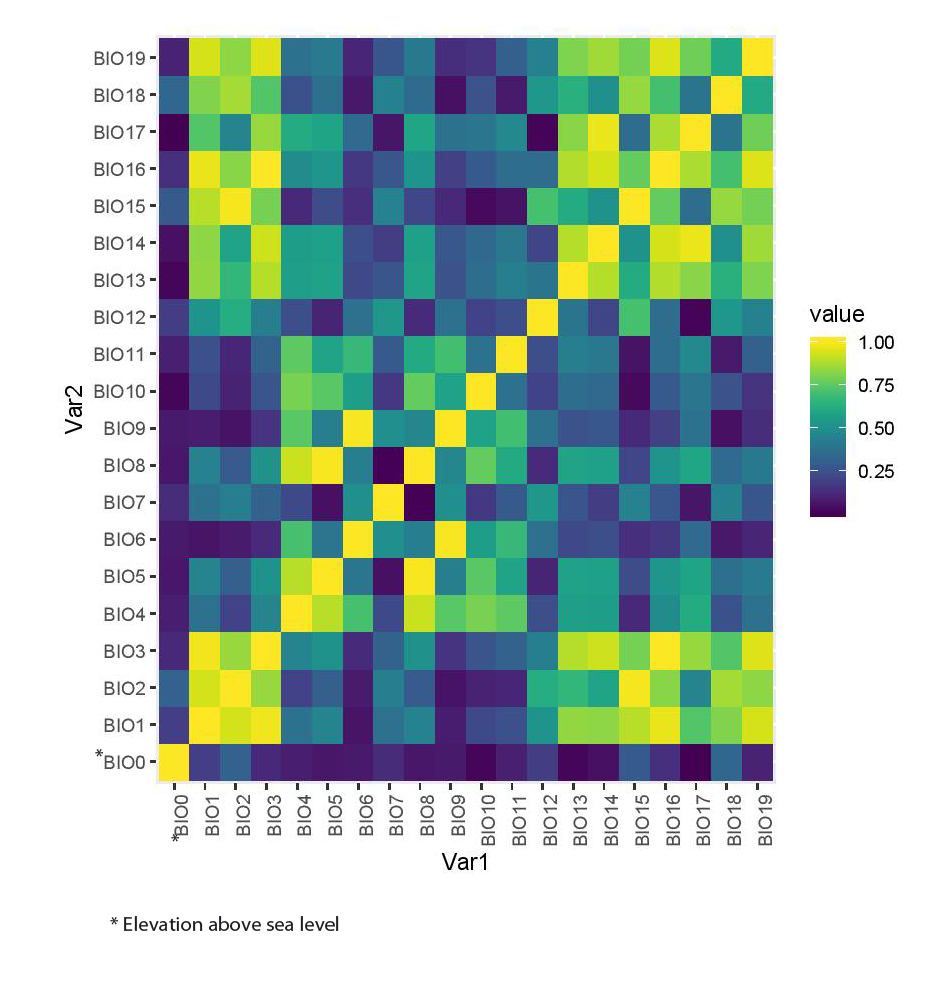

Supplement: Supplementary file 1 — S1 File. Correlation matrix results of the environmental variables collinearity test. S2 File. Correlation matrix Heatmap. S3 File. Evaluation metrics of the Maxent model runs using different parameters and combinations. S4 File. Ae. aegypti prediction model with occurrence points. S5 File. Ae. vexans prediction model with occurrence points. S6 File. Modeling results for Ae. albopictus in Mediterranean Basin countries. S7 File. Ae. albopictus prediction model with occurrence points. S8 File. Ae. caspius prediction model with occurrence points. S9 File. Cx. pipiens prediction model with occurrence points. S10 File. Ae. detritus prediction model with occurrence points. S11 File. Global potential distribution of Ae. vittatus. S12 File. Ae. vittatus prediction model with occurrence points. [file parasite-28-37-olm.zip › parasite200169-olm/S2 File.tif]

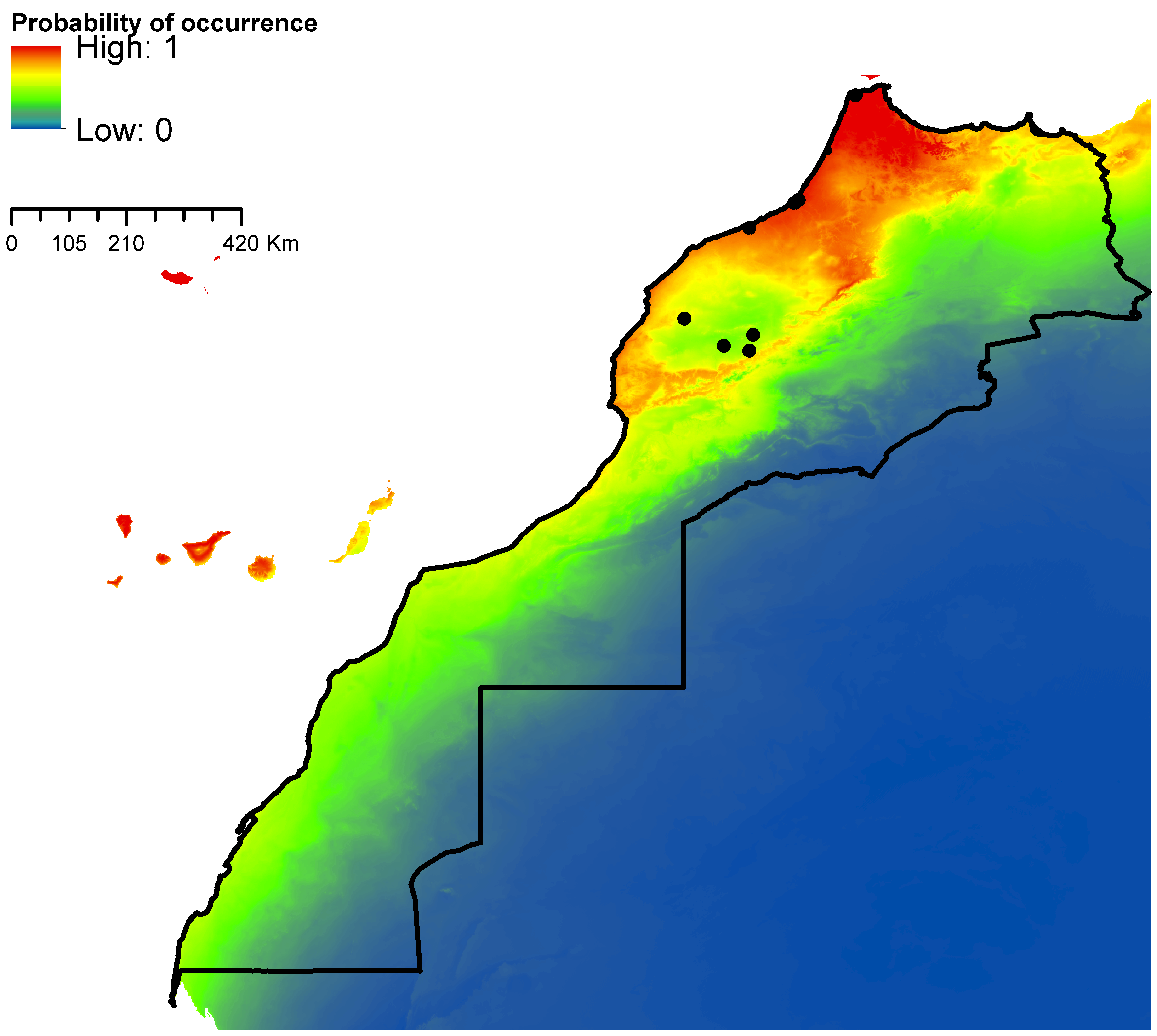

Supplement: Supplementary file 1 — S1 File. Correlation matrix results of the environmental variables collinearity test. S2 File. Correlation matrix Heatmap. S3 File. Evaluation metrics of the Maxent model runs using different parameters and combinations. S4 File. Ae. aegypti prediction model with occurrence points. S5 File. Ae. vexans prediction model with occurrence points. S6 File. Modeling results for Ae. albopictus in Mediterranean Basin countries. S7 File. Ae. albopictus prediction model with occurrence points. S8 File. Ae. caspius prediction model with occurrence points. S9 File. Cx. pipiens prediction model with occurrence points. S10 File. Ae. detritus prediction model with occurrence points. S11 File. Global potential distribution of Ae. vittatus. S12 File. Ae. vittatus prediction model with occurrence points. [file parasite-28-37-olm.zip › parasite200169-olm/S4 File.tif]

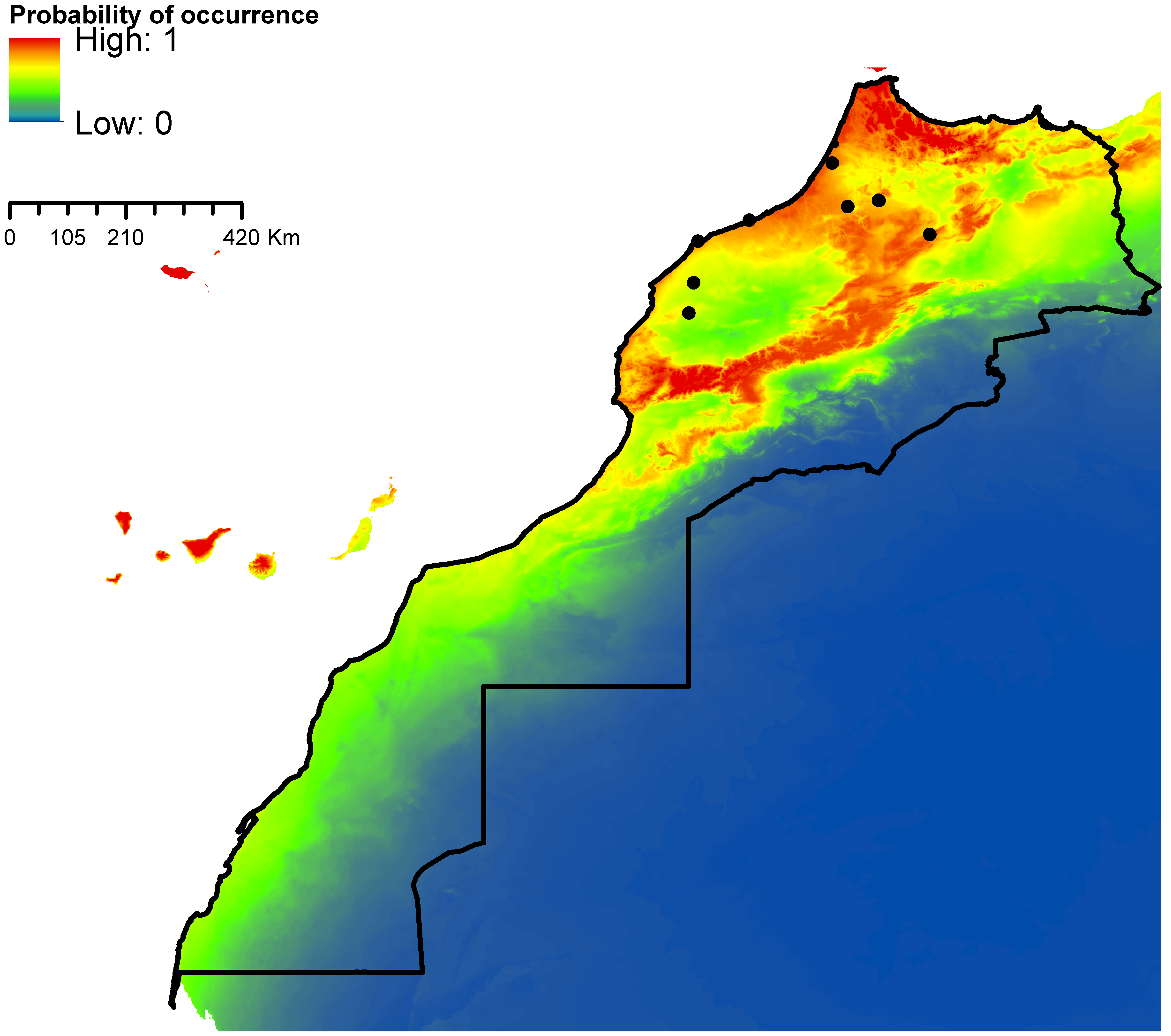

Supplement: Supplementary file 1 — S1 File. Correlation matrix results of the environmental variables collinearity test. S2 File. Correlation matrix Heatmap. S3 File. Evaluation metrics of the Maxent model runs using different parameters and combinations. S4 File. Ae. aegypti prediction model with occurrence points. S5 File. Ae. vexans prediction model with occurrence points. S6 File. Modeling results for Ae. albopictus in Mediterranean Basin countries. S7 File. Ae. albopictus prediction model with occurrence points. S8 File. Ae. caspius prediction model with occurrence points. S9 File. Cx. pipiens prediction model with occurrence points. S10 File. Ae. detritus prediction model with occurrence points. S11 File. Global potential distribution of Ae. vittatus. S12 File. Ae. vittatus prediction model with occurrence points. [file parasite-28-37-olm.zip › parasite200169-olm/S5 File.tif]

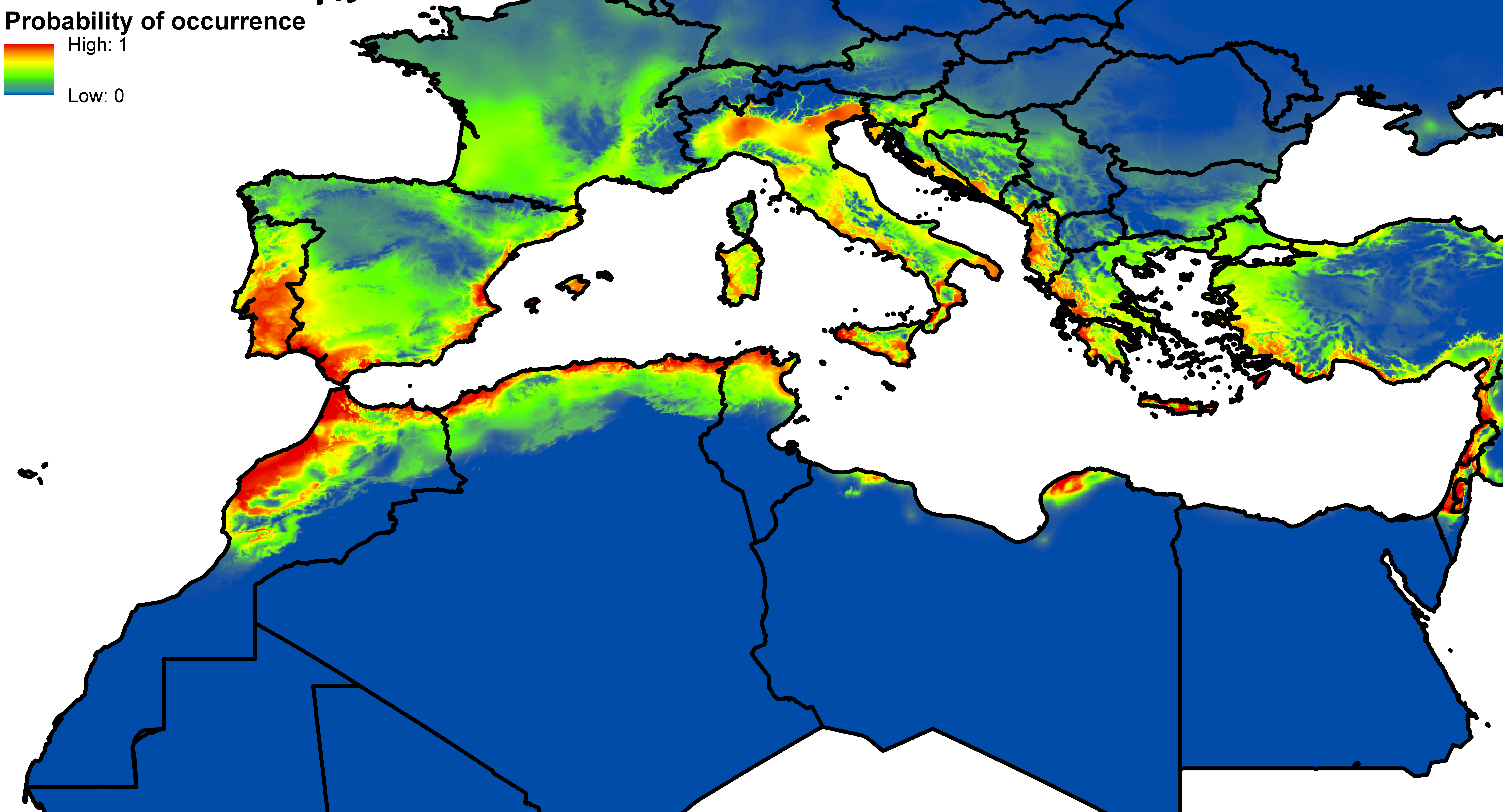

Supplement: Supplementary file 1 — S1 File. Correlation matrix results of the environmental variables collinearity test. S2 File. Correlation matrix Heatmap. S3 File. Evaluation metrics of the Maxent model runs using different parameters and combinations. S4 File. Ae. aegypti prediction model with occurrence points. S5 File. Ae. vexans prediction model with occurrence points. S6 File. Modeling results for Ae. albopictus in Mediterranean Basin countries. S7 File. Ae. albopictus prediction model with occurrence points. S8 File. Ae. caspius prediction model with occurrence points. S9 File. Cx. pipiens prediction model with occurrence points. S10 File. Ae. detritus prediction model with occurrence points. S11 File. Global potential distribution of Ae. vittatus. S12 File. Ae. vittatus prediction model with occurrence points. [file parasite-28-37-olm.zip › parasite200169-olm/S6 File.tif]

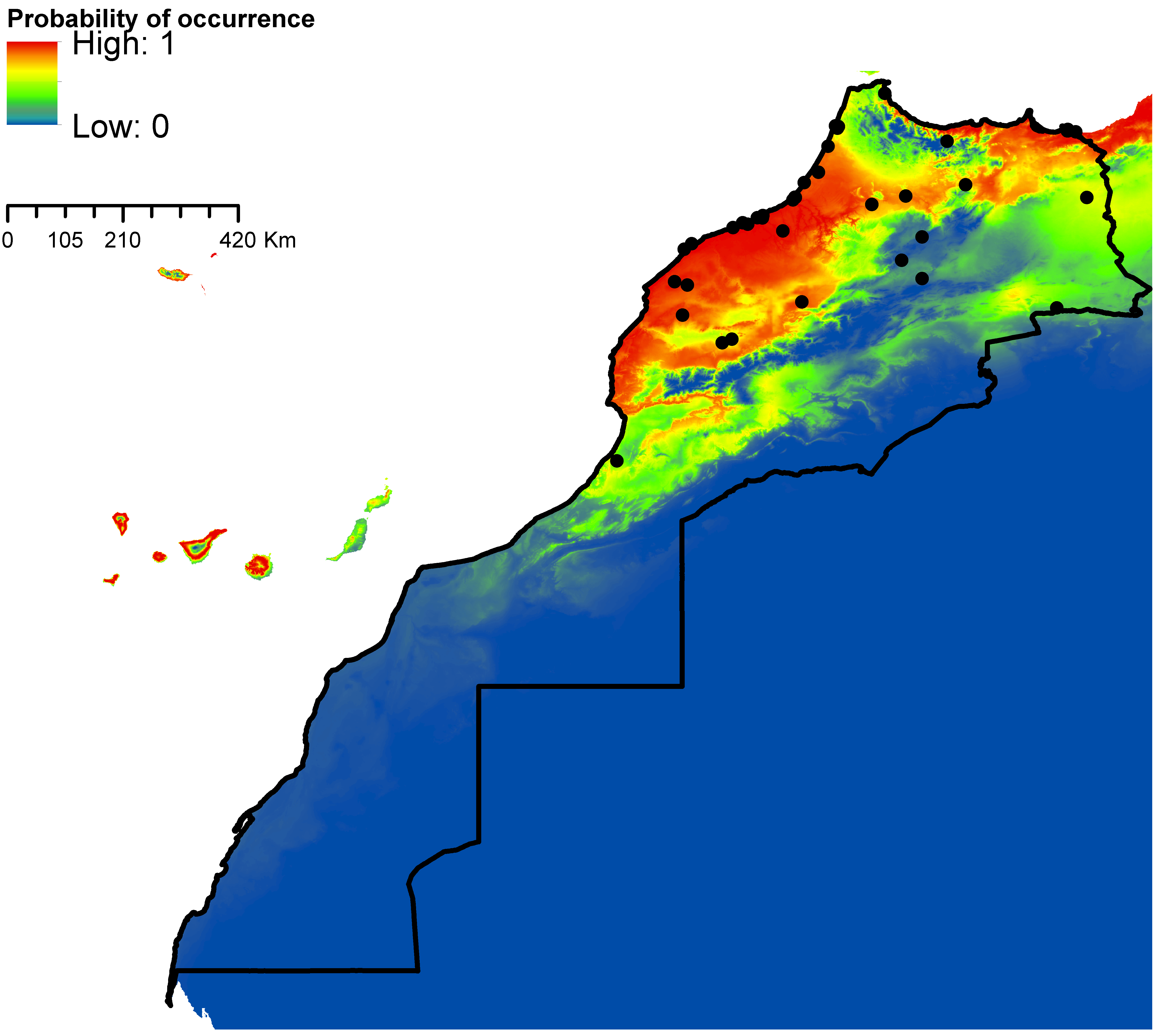

Supplement: Supplementary file 1 — S1 File. Correlation matrix results of the environmental variables collinearity test. S2 File. Correlation matrix Heatmap. S3 File. Evaluation metrics of the Maxent model runs using different parameters and combinations. S4 File. Ae. aegypti prediction model with occurrence points. S5 File. Ae. vexans prediction model with occurrence points. S6 File. Modeling results for Ae. albopictus in Mediterranean Basin countries. S7 File. Ae. albopictus prediction model with occurrence points. S8 File. Ae. caspius prediction model with occurrence points. S9 File. Cx. pipiens prediction model with occurrence points. S10 File. Ae. detritus prediction model with occurrence points. S11 File. Global potential distribution of Ae. vittatus. S12 File. Ae. vittatus prediction model with occurrence points. [file parasite-28-37-olm.zip › parasite200169-olm/S8 File.tif]

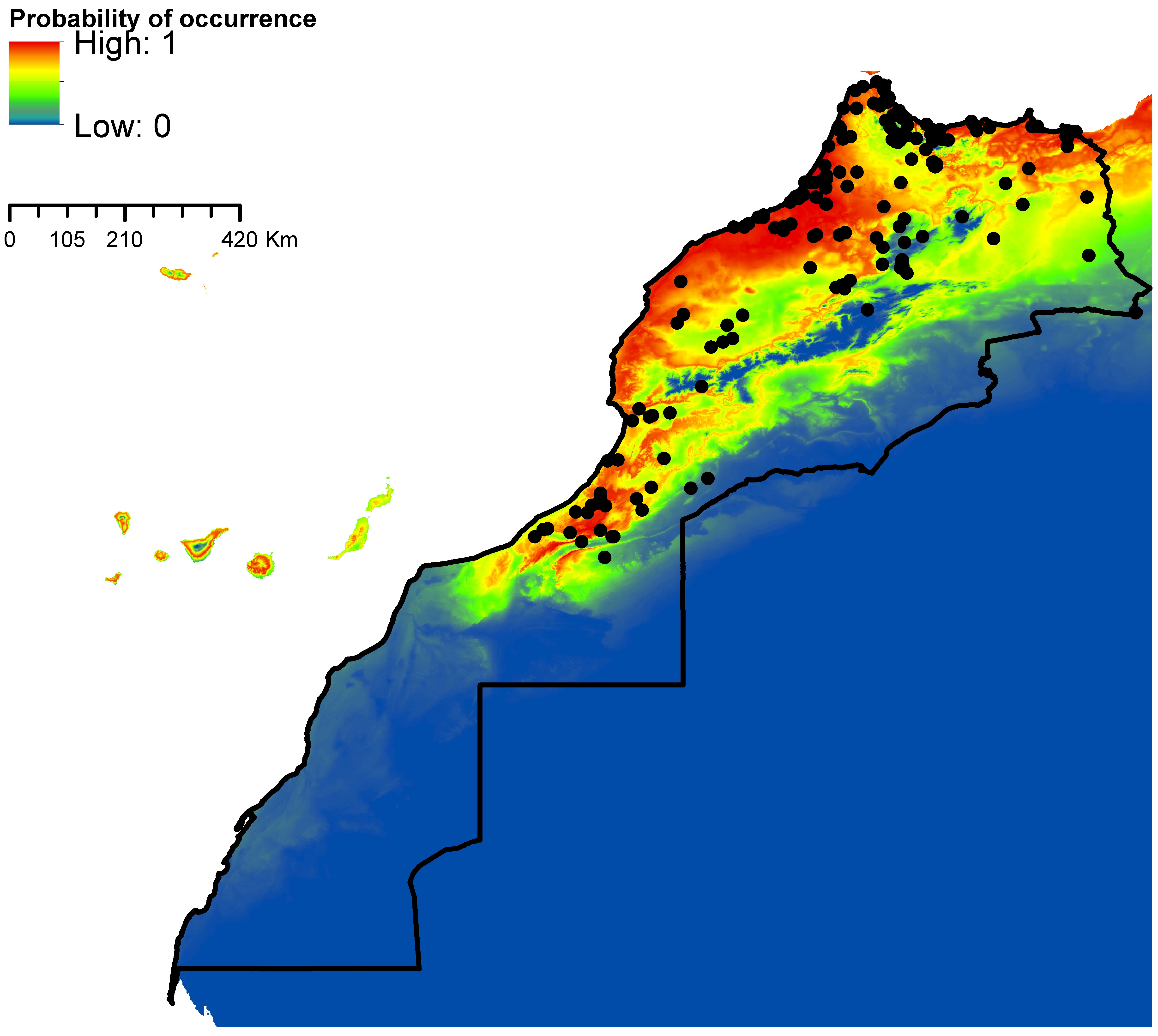

Supplement: Supplementary file 1 — S1 File. Correlation matrix results of the environmental variables collinearity test. S2 File. Correlation matrix Heatmap. S3 File. Evaluation metrics of the Maxent model runs using different parameters and combinations. S4 File. Ae. aegypti prediction model with occurrence points. S5 File. Ae. vexans prediction model with occurrence points. S6 File. Modeling results for Ae. albopictus in Mediterranean Basin countries. S7 File. Ae. albopictus prediction model with occurrence points. S8 File. Ae. caspius prediction model with occurrence points. S9 File. Cx. pipiens prediction model with occurrence points. S10 File. Ae. detritus prediction model with occurrence points. S11 File. Global potential distribution of Ae. vittatus. S12 File. Ae. vittatus prediction model with occurrence points. [file parasite-28-37-olm.zip › parasite200169-olm/S9 File.tif]
